# Responsiveness and convergent validity of QLU-C10D and EQ-5D-3L in assessing short-term quality of life following esophagectomy

**DOI:** 10.1186/s12955-021-01867-w

**Published:** 2021-10-02

**Authors:** Norma B. Bulamu, Ravi Vissapragada, Gang Chen, Julie Ratcliffe, Louise A. Mudge, B. Mark Smithers, Elizabeth A. Isenring, Lorelle Smith, Glyn G. Jamieson, David I. Watson, The Australian Immunonutrition Study Group, Louise A. Mudge, Justin Bessell, Andrew Barbour, Elizabeth A. Isenring, Ahmad Aly, Stephen Archer, Mohammed Ballal, Jessica Barbon, Katie Benton, Melissa Bond, Melissa Berryman, Tim Bright, Richard Cade, Anna Cardamis, Rosemary Carroll, Kara Cashman, Larissa Chan, Brooke Chapman, Sally Chapman, Derek Chen, Jacob Chisholm, Wendy Davidson, Peter Devitt, Cuong Dong, Ra’eesa Doola, Suzanne Edwards, Krishna Epari, Maree Farley, Julie Farrow, Maree Ferguson, David Fletcher, Kerry Forbes, Katherine Fullerton, Philip Game, Susan Gan, David Gotley, Belinda Gout, Jodi Gray, Susan Heaney, Glyn G. Jamieson, Mary Anne Johnson, Megan Johnstone, Sanjeeva Kariyawasam, Jonathan Karnon, Amber Kelaart, Liz Kellett, Erin Kennedy, Rhiannon Krane, Sylvia Lemass, Jenelle Loeliger, Andrew Lord, John Ludbrook, Catherine McFarlane, Michelle McPhee, Selena Yue-Xian Ooi, Leonie Pearce, Kate Pettigrew, Emma Putrus, Georgina Rassias, Alison Shanks, Jon Shenfine, Emma Louise Smith, Lorelle Smith, Justin Singleton, B. Mark Smithers, Rebecca Lindstrom-Sowman, John Spillane, Liliana Sputore, Belinda Steer, Tom Sullivan, Laisa Teleni, Deb Tolcher, Janine Thomas, Sarah Thompson, Tina Thorpe, David I. Watson, Cheryl Watterson, Vanessa Wills, Anita Wilton, Kellie Wright, Tim Wright

**Affiliations:** 1 Flinders Health and Medical Research Institute, College of Medicine and Public Health, Flinders University, Adelaide, South Australia Australia; 2 Department of Surgery, Flinders Medical Centre, Adelaide, South Australia Australia; 3 Centre for Health Economics, Monash Business School, Monash University, Melbourne, VIC Australia; 4 Caring Futures Institute, College of Nursing and Health Sciences, Flinders University, Adelaide, South Australia Australia; 5 Discipline of Surgery, The University of Adelaide, Adelaide, South Australia Australia; 6 Upper GI and Soft Tissue Unit, Academy of Surgery, Princess Alexandra Hospital, Faculty of Medicine, University of Queensland, Brisbane, Australia; 7 Faculty of Health Sciences and Medicine, Bond University, Brisbane, Australia; 8 Department of Gastroenterology and Hepatology, Royal Adelaide Hospital, Adelaide, Australia

**Keywords:** Health related quality of life, QLU-C10D, EQ-5D-3L, Responsiveness, Convergent validity, Ceiling effects, Esophagectomy

## Abstract

**Aim:**

This study assessed the responsiveness and convergent validity of two preference-based measures; the newly developed cancer-specific EORTC Quality of Life Utility Measure-Core 10 dimensions (QLU-C10D) relative to the generic three-level version of the EuroQol 5 dimensions (EQ-5D-3L) in evaluating short-term health related quality of life (HRQoL) outcomes after esophagectomy.

**Methods:**

Participants were enrolled in a multicentre randomised controlled trial to determine the impact of preoperative and postoperative immunonutrition versus standard nutrition in patients with esophageal cancer. HRQoL was assessed seven days before and 42 days after esophagectomy. Standardized Response Mean and Effect Size were calculated to assess responsiveness. Ceiling effects for each dimension were calculated as the proportion of the best level responses for that dimension at follow-up/post-operatively. Convergent validity was assessed using Spearman’s correlation and the level of agreement was explored using Bland–Altman plots.

**Results:**

Data from 164 respondents (mean age: 63 years, 81% male) were analysed. HRQoL significantly reduced on both measures with large effect sizes (> 0.80), and a greater mean difference (0.29 compared to 0.16) on QLU-C10D. Both measures had ceiling effects (> 15%) on all dimensions at baseline. Following esophagectomy, ceiling effects were observed with self-care (86%), mobility (67%), anxiety/depression (55%) and pain/discomfort (19%) dimensions on EQ-5D-3L. For QLU-C10D ceiling effects were observed with emotional function (53%), physical function (16%), nausea (35%), sleep (31%), bowel problems (21%) and pain (20%). A strong correlation (r = 0.71) was observed between EQ-5D-3L anxiety and QLU-C10D emotional function dimensions. Good agreement (3.7% observations outside the limits of agreement) was observed between the utility scores.

**Conclusion:**

The QLU-C10D is comparable to the more widely applied generic EQ-5D-3L, however, QLU-C10D was more sensitive to short-term utility changes following esophagectomy. Cognisant of requirements by policy makers to apply generic utility measures in cost effectiveness studies, the disease-specific QLU-C10D should be used alongside the generic measures like EQ-5D-3L.

*Trial registration*: The trial was registered with the Australian New Zealand Clinical Trial Registry (ACTRN12611000178943) on the 15th of February 2011.

**Supplementary Information:**

The online version contains supplementary material available at 10.1186/s12955-021-01867-w.

## Introduction

Esophageal cancer is the 8th most common cancer in the world with a global incidence of 6.7 per 100,000 and a generally poor prognosis [1, 2]. There are two major subtypes, squamous cell carcinoma (ESCC) and adenocarcinoma (EAC). Overall 5-year survival rates for populations developing these cancers are reported to be 17–20% [3]. The majority of patients present with vague symptoms including difficulty in swallowing and weight loss, with diagnosis obtained using endoscopy and biopsy. Treatment for EAC depends on TNM staging (T = tumour growth through tissue, N = nodal involvement and M = metastatic involvement). Uni-modality treatment involves surgery or endoscopic resection, dual modality involves chemotherapy and radiation while tri-modality involves surgery, chemotherapy and radiation. Uni-modality treatment is preferred for the earliest stages of cancer. Surgery for EAC or esophagectomy is an invasive operation involving access to thorax and abdomen. Parts of the esophagus and stomach are resected along with adjacent lymph nodes followed by anastomosis of the reshaped stomach to the remaining esophagus [4]. Overall, treatment for esophageal cancer is expensive, resource intensive, and carries a high morbidity and quality of life detriments [5, 6]. It is important to understand the QoL experienced by patients undergoing treatment to guide tailoring of interventions and patient information but also to inform health funding decisions.

A plethora of instruments exist for the assessment of quality-of-life outcomes, including both generic and condition-specific measures, which can be preference based, also refered to as mulit-attribute utility instruments (MAUIs) or non-preference based. Preference-based instruments facilitate the calculation of quality-adjusted life years (QALYs) and are therefore suitable for application in cost-utility analysis (CUA). Decision making bodies including the National Institute for Health and Care Excellence (NICE) in the United Kingdom, and the Pharmaceutical Benefits Advisory Committee (PBAC) and Medical Services Advisory Committee (MSAC) in Australia, require the use of generic MAUIs such as the EuroQol 5-dimensions EQ-5D (EQ-5D-3L and EQ-5D-5L) in submissions of economic evaluations of cost effectiveness evidence [7, 8]. However, it has been argued that generic measures are not as sensitive to changes in quality of life, particularly in cancer, and thus may not adequately reflect quality of life detriments or gains in these conditions which would ultimately affect any QALY estimations undertaken [9, 10]. This has led to the development of cancer-specific MAUIs such as the European Organization for Research and Treatment of Cancer Eight dimensions (EORTC-8D) and EORTC Quality of Life Utility Measure-Core 10 dimensions (QLU-C10D), which are both derived from the non-preference-based EORTC Core Quality of Life Questionnaire (EORTC QLQ-C30) [11, 12].

The EORTC-8D has eight dimensions obtained from 10 of the 30 items of QLQ-C30. It has four functional scales including physical functioning, role functioning, emotional functioning, and social functioning, and four symptom scales of pain, fatigue and sleep disturbances, nausea and lastly constipation and diarrhoea. This instrument generates a total of 81,920 health states [12]. Health states were valued by a general population sample in the United Kingdom using the time trade-off valuation method [13]. The newer MAUI, QLU-C10D, is an extension of the work done in developing the EORTC-8D [11, 14]. It has four functional scales and six symptom scales obtained from 13 out of the 30 items of QLQ-C30 [11]. The functional scales are similar but the symptom scales also include appetite and bowel problems instead of constipation and diarrhoea. QLU-C10D generates 1,048,576 health states and a value set generated from an Australian general population sample using discrete choice experiments methodology [14]. Although both measures are obtained from QLQ-C30, they are different in some respects. Emotional functioning, physical functioning, fatigue, nausea and bowel problems are obtained from the same QLQ-C30 items for both measures; however, physical functioning has four levels in QLU-C10D and five levels in EORTC-8D. Role functioning, social functioning and pain are obtained from different items for both measures and QLU-C10D has two more dimensions, sleep and appetite which are not present in the EORTC-8D [11].

Given both preference-based generic and cancer-specific MAUIs are available to measure and value health related quality of life (HRQoL) in cancer patient populations, it is important to understand the relative psychometric performance of these instruments in clinical trials. Two studies that compared the generic EQ-5D-3L and EORTC-8D found that although both measures had similar discriminatory power, the calculated QALYs based on EQ-5D-3L were significantly lower than those obtained using EORTC-8D [9, 15]. This inconsistency could lead to substantial opposite conclusions as to whether an intervention should be regarded as cost-effective or not, and this is critical for resource intensive and high morbidity interventions like esophagectomy.

Unlike the EORTC-8D, there is a lack of longitudinal evidence on the comparisons of psychometric properties between the newly developed QLU-C10D and EQ-5D-3L or any generic MAUIs. This paper aimed to contribute to the literature by assessing the responsiveness and convergent validity of the newly developed cancer-specific QLU-C10D relative to the generic EQ-5D-3L in the context of short-term quality of life/utilities after esophagectomy for esophageal cancer.

## Methods

### Sample

This analysis was undertaken from a pooled sample of patients participating in a randomised control trial, the details of which are reported elsewhere [16]. Briefly, patients were randomised into four groups to receive an immunonutrition supplement or standard nutrition without added immunonutrients; (1) before but not after; (2) after but not before; (3) both before and after; and (4) none before or after esophagectomy. Quality of life was assessed seven days before and 42 days after esophagectomy. As briefly stated in an earlier publication there were no significant differences in quality of life or clinical outcomes between the groups before and after esophagectomy [16]. In this paper data was analysed as a pooled sample including all patients with both baseline (pre-operative) and follow-up (post-operative) quality of life scores on the EORTC QLQ-C30 and EQ-5D-3L measures. Patients were excluded from this analysis if they had any missing value of these two quality of life measures at any timepoint.

### Quality of life assessment

Quality of life was assessed using the self-administered and widely validated generic utility-based EQ-5D-3L [17] and the cancer specific EORTC-QLQ-C30 [18]. The EQ-5D-3L descriptive system comprises five dimensions: mobility, self-care, usual activities, pain/discomfort and anxiety/depression with 3 levels for each dimension: no problems, some problems, and extreme problems. The EQ-5D-3L was scored using an Australian-specific tariff [19]. The QLQ-C30 has one global HRQoL scale, five functional scales (physical, role, emotional, cognitive, social), three symptom scales (fatigue, nausea or vomiting, pain) and six single items (sleeping disorders, appetite loss, dyspnoea, diarrhoea, constipation, and financial problems). Each item has four alternative responses (1- not at all; 2- a little; 3-quite a bit; 4-very much). Responses to the QLQ-C30 were mapped onto the cancer specific preference based QLU-C10D to generate utility scores using an Australian-specific scoring algorithm developed by Norman et al. [14] for this purpose. Research has showed that country-specific value sets, where available, are preferable when evaluating interventions in that country/region [20, 21]. As such, although both EORTC-8D and QLU-C10D utilities can be derived from the QLQ-C30, QLU-C10D was preferable in this context because it’s health states have been valued by an Australian general population sample.

### Data analysis

Data was analysed using Stata (StataCorp, College Station, TX, USA) [22]. Normally distributed data was analysed with one-way analysis of variance (ANOVA), and Kruskal–Wallis H test was used for analysis of non-normally distributed data. A p-value < 0.05 was considered statistically significant.

#### Mean difference

Utility scores for EQ-5D-3L [19] and QLU-C10D [14] were generated based on Australian general population scoring algorithms pertaining to each instrument. Basic descriptive statistics including means, medians and ranges were compared for each instrument at baseline and follow-up. The clinically important mean difference (MCID) for EQ-5D-3L when used in populations with cancer varies from 0.07—0.12 [23]. A change of > 10 with the EORTC QLQ-C30 is considered clinically relevant and > 20 as strongly relevant [24].

#### Responsiveness

To assess responsiveness (i.e. the ability of an instrument to detect changes in response to esophagectomy in this study), two statistical tests were applied, including the Standardized Response Mean (SRM) and Effect Size (ES) [25]. They are calculated as ES = ratio of the mean change to the standard deviation of scores at baseline statistic and the SRM = ratio of the mean change to the standard deviation of that change. For ES scores, the recommended minimum effect size = 0.41, moderate effect = 1.15 and strong effect = 2.70 [26]. SRM scores of < 0.20 = trivial effect, 0.20– < 0.50 = small effect, 0.50–0.80 = moderate effect, > 0.80 = large effect [27].

#### Ceiling effects

Ceiling effect is a measure of how accurately an instrument measures the intended domain by considering the proportion of respondents who achieve the highest level of the domains or the highest score of the instrument [28]. Ceiling effects are present ‘if more than 15–20% of respondents achieved the best possible score’ [29, 30]. Ceiling effects were calculated at both baseline and follow-up. The ceiling effect for EQ-5D-3L was calculated as the proportion of ‘no problem’ responses on each dimension and the proportion of ‘no problem’ in all dimensions. Similarly, for the QLU-C10D the ceiling effect was calculated as the proportion of level 1 (no trouble/limitation) on each dimension as well as on all dimensions. Ceiling effects were further explored by selecting those reporting full health in one instrument to see what they report in the other instrument. Lower ceiling effects suggest greater discriminant ability.

#### Convergent validity

Convergent validity was explored between dimensions measuring similar constructs on both measures such as mobility and physical function, pain/discomfort and pain [31, 32]. Correlations were classified as very weak (r = 0–0.2), weak (r = 0.2–0.4), moderate (r = 0.4–0.7), strong (r = 0.7–0.9) or very strong (r = 0.9–1.0) [27].

#### Agreement

The limits of agreement between the instruments were explored using Bland–Altman plots [33, 34]. Good agreement was demonstrated by less than 5% of points being outside of the limits of agreement (LOA).

## Results

### Demographics

164 of the original cohort of 276 patients completed both quality of life questionnaires before and after surgery and were included in the analysis presented in this study. 112 patients were excluded as they only had one set of quality of life data. There were no significant differences in demographic characteristics between patients included (n = 164) in this analysis vs. those excluded (n = 112) for all but two variables. More patients in the excluded group had TNM stage IIIc/4 (14% compared to 4%) and underwent a thoracoscopic esophagectomy (66% compared to 54%)—see Additional file 1: Table A1. However, these differences did not translate into differences in HRQoL outcomes, see Additional file 1: Table A2.

**Table 1:** Demographic and clinical characteristics

Characteristic	Statistics		
	n	Mean (sd)	Median (IQR)
*Demographic variables*			
Age	164	62.9 (7.9)	63.6 (58.4, 67.9)
Male Gender (n/%)	133 (81)		
Alcohol (n/%)	106 (66)		
Smoking (n/%)	22 (13)		
*Clinical variables*			
Hospital length of stay (days)	164	17 (15)	13 (11, 18)
ICU length of stay (days)	164	4 (4)	3 (1, 5)
Blood loss	163	268.1 (256.1)	200 (0, 400)
Blood transfusion (units)	164	0.3 (2.0)	0 (0,0)
Tumour length (cm)	114	3.9 (2.7)	3.3 (2, 5.5)
Total pack years	164	16 (18)	15 (0, 29)
Hospital length of stay > 10 days (n/%)	133 (81)		
Tumour length > 3 cm (n/%)	58 (51)		

	(n/%)
*Co-morbidities*	
Hypertension	62 (36)
Diabetes	21 (13)
Respiratory	33 (20)
Cardiac	27 (17)
*Treatment*	
Preoperative radiotherapy	67 (41)
Preoperative chemotherapy	129 (79)
*ASA score*	
1 or 2	111 (68.1)
3	52 (31.9)
*Pathological T staging*	
0/1/1a/1b/Tis	72 (44)
2/3	92 (56)
*TNM staging*	
0/IA/IB	75 (46)
II/IIB/IIIA/IIIB	82 (50)
IIIC/4	7 (4)
*Procedure type*	
Open chest and abdominal approach	76 (46)
Hybrid (thoracoscopic)	88 (54)

*ICU* intensive care unit, *IQR* interquartile range, *SD* standard deviation

**Table 2:** Descriptive statistics including mean difference and effect size

	EQ-5D-3L	QLU-C10D	EORTC summary score
Pre-operative mean (sd)	0.85 (0.15)	0.81 (0.16)	84.3 (13.1)
Post-operative mean (sd)	0.69 (0.16)	0.52 (0.22)	62.5 (17.9)
Mean difference (95% CI)	0.16 (0.13, 0.19)	0.29 (0.26, 0.32)	21.6 (18.9, 24.4)
*p* value^a1^	0.000*	0.000*	0.000*
Standard effect size (ES)^b2^	1.08	1.53	1.38
Standard response mean (SRM)^c3^	0.84	1.37	1.22

^a1^ttest difference in mean between baseline (7 days before esophagectomy) and follow-up (42 days after esophagectomy)

^b2^Cohen’s d effect size calculation, recommended minimum effect size = 0.41, moderate effect = 1.15 and strong effect = 2.70

^c3^Standard Response Measure (ratio of the mean change to the standard deviation of that change), < 0.20 = trivial effect, 0.20– < 0.50 = small effect, 0.50–0.80 = moderate effect, > 0.80 = large effect

*Statistically significant values at < 0.05

Table 1 summarises the demographic characteristics of the study sample. Participants were predominantly male (81%), mean age was 63 years, with a history of alcohol consumption (66%). The commonest surgical technique (54%) was a “hybrid” esophagectomy entailing and open abdominal phase, thoracoscopic chest phase and anastomosis in the left neck. Most patients had preoperative chemotherapy (79%), and length of the hospital stay was more than 10 days (81%) for most patients.

### HRQoL mean difference and effect size

HRQoL reduced between baseline (7 days before) and follow-up (42 days) after esophagectomy on all measures, 0.85–0.69 on EQ-5D-3L, 0.81–0.52 with QLU-C10D, and 84.3 to 62.5 with QLQ-C30 and these changes were statistically significant (Table 2). The mean score differences for EQ-5D-3L and QLQ-C30 exceeded their respective MCID at 0.16 and 21.6 respectively. The mean difference for QLU-C10D was 0.29 but the MCID for this measure has not yet been established. As such, the MCID for QLQ-C30 was reported and used as a reference. Moderate effect was detected for all measures using ES (> 1.15 but < 2.70) and strong effect using SRM (> 0.80). QLU-C10D was a more responsive measure compared to EQ-5D-3L with a larger ES (1.53 compared to 1.08) and SRM (1.37 compared to 0.84).

### Ceiling effects

**Fig 1:**
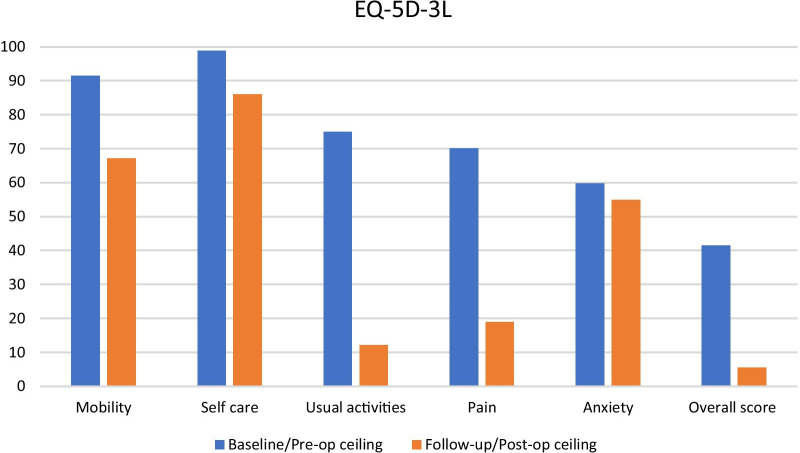
EQ-5D-3L ceiling effect at baseline and follow-up

Distribution of the scores was similar for both measures at baseline and follow-up. However, a clustering of EQ-5D-3L indices at the upper level with a gap between 1 and the lower levels was observed at baseline. Ceiling effects (> 15%) were observed on the EQ-5D-3L for all dimensions at baseline and similarly after esophagectomy except for the usual activities dimension (Fig. 1). The self-care dimension showed the greatest ceiling effect at both baseline (99%) and follow-up (86%). Usual activities and pain showed the greatest (> 50%) reduction in ceiling effects between baseline and follow-up.

**Fig 2:**
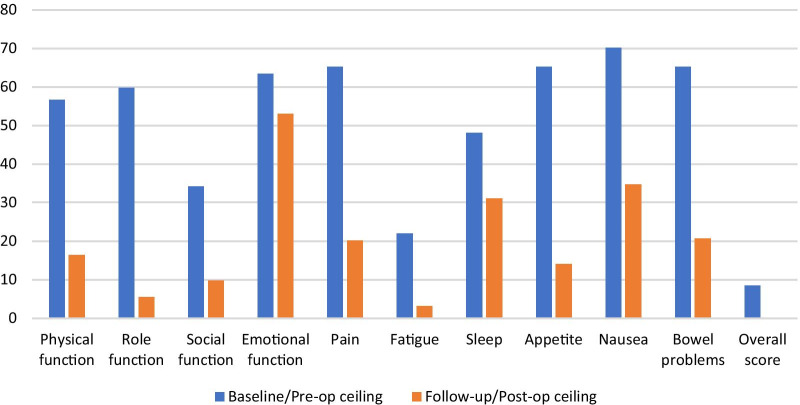
QLU-C10D ceiling effects at baseline and follow-up

For QLU-C10D ceiling effects were observed for all dimensions pre-operatively but only with emotional function (53%), physical function (16%), nausea (35%), sleep (31%), bowel problems (21%) and pain (20%) following esophagectomy (Fig. 2). Nausea (70%) and emotional function (53%) had the highest ceiling effects at baseline and follow-up respectively. Role function and appetite showed the greatest (> 50%) reduction in ceiling effect between baseline and follow-up.

At baseline 13 (8%) respondents reported full health on both measures, while 55 (34%) reported full health on EQ-5D-3L but not QLU-C10D. Nine respondents (5.5%) reported full health on EQ-5D-3L but none reported full health on QLU-C10D at follow-up. Table 3 summarises QLU-C10D responses for the respondents who reported full health on EQ-5D-3L but not QLU-C10D at baseline (55) and at follow-up (9). Patients reporting full health with EQ-5D-3L still had problems when the disease specific measure was used, particularly with social function (51%) and fatigue (67%) at baseline and fatigue (78%) at follow-up where majority had less than the highest domain score.

**Table 3:** Distribution of QLU-C10D responses for participants reporting full health on EQ-5D but not on QLU-C10D

Level	Functional domains				Symptoms					
	Physical	Role	Social	Emotional	Pain	Fatigue	Sleep	Appetite	Nausea	Bowel
Baseline n = 55^a^										
1 (best)	69	80	36	80	87	25	65	73	84	65
2	29	18	**51**	20	13	**67**	35	24	16	33
3	2	2	9	0	0	7	0	4	0	0
4 (worst)	0	0	4	0	0	0	0	0	0	2
Follow-up n = 9^a^										
1 (best)	44	56	67	89	78	0	56	33	56	67
2	33	33	22	11	22	**78**	22	22	22	33
3	22	11	11	0	0	11	22	22	22	0
4 (worst)	0	0	0	0	0	11	0	22	0	0

^a^55 respondents reported full health on EQ-5D but not QLU-C10D at baseline and 9 respondents reported full health on EQ-5D but not on QLU-C10D at follow-up

### Convergent validity

Results of the correlation between measures for both utility and dimensions scores are reported in Table 4. QLU-C10D and EQ-5D-3L utility score were strongly correlated (r = 0.71). Correlation between the EQ-5D-3L utility score and QLU-C10D functional domains were moderate (r > 0.4) but weak correlations (r < 0.4) were observed with the symptom domains. At the dimension level, anxiety/depression was strongly correlated (r = 0.71) with emotional function on the QLU-C10D while moderate correlations were observed for mobility and physical function (r = 0.6), usual activities with role function (r = 0.68), social function (r = 0.54), and fatigue (r = 0.41) as well as pain/discomfort with pain (r = 0.55). Very weak correlations were observed for pain/discomfort with role function (r = 0.18), nausea (r = 0.17) and bowel problems (r = 0.2), mobility with pain (r = 0.19) and nausea (r = 0.18), as well as usual activities with pain (r = 0.2) and personal care with fatigue (r = 0.18).

**Table 4:** Correlation between QLU-C10D and EQ-5D domains at follow-up

	EQ-5D utility score	Mobility	Self care	Usual activities	Pain/discomfort	Anxiety/depression
QLU-C10D utility score	0.7138	− 0.4804	− 0.2754	− 0.5857	− 0.2402	− 0.4608
Function domains						
Physical function	− 0.5532	0.6022	0.3468	0.4594		0.2783
Role function	− 0.6132	0.4161	0.2535	0.6758	0.1767	0.2668
Social function	− 0.5715	0.3075	0.2950	0.5387		0.3766
Emotional function	− 0.6009	0.2474		0.3995		0.7113
Symptom domains						
Pain	− 0.3190	0.1886		0.1966	0.5517	
Fatigue	− 0.4716	0.3265	0.1755	0.4095		0.3155
Sleep	− 0.3385	0.2567	0.1926	0.2763		0.3241
Appetite	− 0.3470	0.2106		0.2769		0.3235
Nausea	− 0.3790	0.1829		0.2721	0.1746	0.3561
Bowel problems	− 0.2231				0.1990	0.2780

Correlations between dimensions were explored using patient responses at follow-up (42 days after esophagectomy). Only significant correlations (at < 0.05) are reported. Correlations were classified as very weak (r = 0–0.2), weak (r = 0.2–0.4), moderate (r = 0.4–0.7), strong (r = 0.7–0.9) or very strong (r = 0.9–1.0)

### Agreement

**Fig 3:**
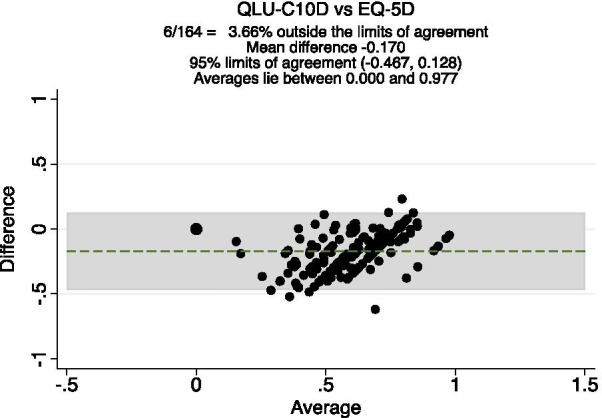
Bland–Altman plot of QLU-C10D and EQ-5D-3L at follow-up

The Bland Altman plot (Fig. 3) showed a small mean difference and good agreement between QLU-C10D and EQ-5D-3L utility scores as only 3.7% observations were outside the limits of agreement.

## Discussion

The clinical trial [16] underpinning the current study compared a homogenous group of patients with esophageal cancer (Table 1) who underwent surgical resection and received different regimens of nutritional support. As the outcomes from the trial showed no differences in clinical and quality of life outcomes between the different nutritional support regimens, the data was analysed as a pooled sample in this current paper. This analysis assessed the responsiveness and convergent validity of the cancer specific QLU-C10D and generic EQ-5D-3L for measurement of short-term HRQoL outcomes following esophagectomy.

As expected, both measures showed statistically significant reductions in HRQoL following surgery (Table 2). Utility decrements of 0.16 and 0.29 were observed with EQ-5D-3L and QLU-C10D respectively, and that of EQ-5D-3L was clinically significant [23]. As the QLU-C10D is a relatively new instrument, there has been no MCID reported in the literature yet, however, the reduction in QLQ-C30 score of 21.6 was clinically significant [24]. On comparing the EQ-5D-3L and QLU-C10D, the EQ-5D-3L mean utility score was greater than QLU-C10D by 0.04 at baseline and 0.17 at follow-up. The small difference at baseline but not at follow-up suggests a high degree of convergence for mild health states but not for severe states such as after esophagectomy, where the limitations of a generic measure become apparent, and a more sensitive disease specific measure is preferred.

Both instruments reported a large effect size with QLU-C10D being larger than the EQ-5D-3L. Most studies with EQ-5D-3L have reported low to moderate effect size [35]. However, studies with large expected changes in health status such as after surgery have reported large effect size [36–38]. Although both EQ-5D-3L and QLU-C10D are on the 0–1 QALY scale (where 0 is equivalent to being dead and 1 is full health), a much larger effect size was observed with QLU-C10D. This observation is similar to other studies comparing disease specific and generic measures where disease specific measures have much larger effect sizes or show greater responsiveness [39]. This is because the disease specific measures, unlike the generic, assess domains of quality of life that are of greatest importance to the condition.

For both EQ-5D-3L and QLU-C10D, ceiling effects were observed for all dimensions at both pre- and post-operative, with higher ceiling effects observed with EQ-5D-3L (Figs. 2 and 3). The highest ceiling effect for EQ-5D-3L was with self-care at both timepoints and the highest for QLU-C10D was nausea symptoms pre-operative and emotional function after esophagectomy. Ceiling effects of the EQ-5D-3L have been reported in several patient populations but the levels observed here were higher than reported in other studies among similar populations [40, 41]. In addition, the proportion reporting the best score for all dimensions (or full health) was much lower with the QLU-C10D at both timepoints. Yet patients reporting full health with EQ-5D-3L still reported problems when the disease-specific measure was used, particularly with social function and fatigue at baseline and fatigue at after esophagectomy (Table 4). These two dimensions are among four key bolt-on dimensions that been suggested to the current EQ-5D classification system [42]. Our findings are similar to Lorgelly et al. [9] who showed that EQ-5D-3L was less sensitive to fatigue impairment when compared to the disease specific EORTC-8D. Although EQ-5D-3L had a large effect size and demonstrated a clinically meaningful change in utility scores, the high ceiling effects suggest low discriminant ability. This means that the EQ-5D-3L is not as capable of identifying all or most of the change in HRQoL and distinguishing health states in this population. To address such ceiling effects generally experienced with EQ-5D-3L, a 5-level version of the instrument, EQ-5D-5L was developed [43], however, no studies are available comparing it to the QLU-C10D as yet. In addition, increasing the number of response levels alone may not be sufficient to improve the sensitivity of EQ-5D if the key dimensions are not covered. In their analysis investigating gaps in the EQ-5D descriptive system (using the EQ-5D-5L) Chen and Olsen argue for an extended health state classification system that includes four bolt-on dimensions namely vitality, sleep, social relationships and community connectedness [42].

Strong correlations were observed between QLU-C10D and EQ-5D-3L utility scores. The EQ-5D utility score was moderately corelated with all functional domains but weakly correlated with the symptom domains of the QLU-C10D. This is not suprising as unlike QLU-C10D, the EQ-5D measures function and no cancer specific symptom. As such, QLU-C10D is a preferred measure for application in clinical trials and studies in populations with cancer. Similar to other studies that have assessed convergent validity [9], domains that assessed similar constructs showed a strong or moderate correlation such as emotional function and physical function on the QLU-C10D with anxiety/depression and mobility on the EQ-5D-3L respectively.

The limitation of our study is the reduced sample size which resulted from excluding nearly 41% of patients who participated in the original RCT. However, excluding these patients is unlikely to have impacted the results as there were no significant differences in HRQoL outcomes between patients included in this analysis and those that were excluded.

From this analysis, both QLU-C10D and EQ-5D-3L are suitable for economic evaluations assessing interventions following esophagectomy. However, because the QLU-C10D is more responsive to change, QALYs calculated based on QLU-C10D utility scores should be used when comparing interventions in cancer. The variation in mean utility differences from these 2 instruments (0.16 for EQ-5D-3L vs 0.29 for QLU-C10D) translates into a difference in the utility gained from each of them. Although such a difference in utility would normally be treated the same in modelling studies (and only reported in the sensitivity analysis), the choice of preference based instruments influences the CUA results [44]. The major implication is that a decision maker might potentially not fund a treatment strategy, based on a less sensitive tool, when in reality the treatment is worth funding. This is especially useful in clinical conditions like cancer, where the disease specific measure is more sensitive and provides additional differentiation between treatments [9, 11]. For these studies the QLQ-C30 and subsequently QLU-C10D can be applied as a complement to but also as a substitute to the generic measure in assessing HRQoL. Developers of this algorithm have argued that in cases where the QLU-C10D utility scores are correlated with and as sensitive and responsive as those of the generic MAUIs, then QLU-C10D can substitute the generic MAUI [11]. However, using a generic measure, unlike the disease-specific measure, allows for generalisability and comparison of interventions between disparate populations. The generic utilities provide a benchmark comparison for decision-makers, such as PBAC and NICE who are charged with making allocative decisions across a range of pharmaceuticals or diseases within health systems. Yet where only the condition specific utilities have been used, comparisons can still be made because they facilitate the calculation of QALYs which allows for these types of comparison to be made as outcomes are measured on a common QALY scale (although the content of the measures themselves may be quite different between generic and condition specific measures).

Therefore, cognisant of requirements by policy makers to apply generic utility measures in cost effectiveness studies, the disease-specific QLU-C10D could be used alongside the generic measures like EQ-5D-3L. Some studies have applied mapping algortihms where a disease specific measure is mapped onto a generic measures [45, 46]. However, mapping algorithms assume that the generic measure is sensitive to changes in the target population, an assumption that is rarely tested. They are also often of variable quality and can have poor prediction ability as a consequence [46, 47]. With the advent of condition specific utility algorithms, it is now possible and preferable to calculate QALYs directly from the condition specific measure. It is also notable that in Australia the EQ-5D is not mandated for use in economic evaluation studies (unlike NICE in the UK for example), so there are choices to be made and QALYs from condition specific measures can still be used.

## Conclusion

The findings from this study suggest that the newer QLU-C10D is comparable to the more widely applied generic EQ-5D-3L. However, EQ-5D-3L may not be sufficient in isolation when assessing short-term HRQoL following esophagectomy and should be combined with a condition specific measure. Cognisant of requirements by policy makers such as NICE in the UK and PBAC and MSAC in Australia to apply generic utility instruments in cost effectiveness studies, we recommend the application of a condition specific utility instrument alongside the generic instrument. In this way the sensitivity of a cost-effectiveness assessment decision can be determined by considering utility estimates generated from both a condition specific and a generic measure.

## Supplementary Information

**Additional file 1**. Supplementary material

## Data Availability

The datasets used and/or analysed during the current study are available from the corresponding author on reasonable request.

## Abbreviations

CEA: Cost effectiveness analysis
CUA: Cost utility analysis
EORTC: European Organization for Research and Treatment of Cancer
EORTC QLQ-C30: EORTC Quality of Life Questionnaire Core 30 item
EORTC QLU-C10D: EORTC Quality of Life Utility Measure-Core 10 dimensions
EORTC-8D: EORTC Eight dimensions
EQ-5D: EuroQol 5 dimensions
HRQoL: Health related quality of life
LOA: Limits of agreement
MAUIs: Multi-attribute utility instruments
MSAC: Medical Services Advisory Committee
NICE: National Institute for Health and Care Excellence
PBAC: Pharmaceutical Benefits Advisory Committee
QALYs: Quality adjusted life years
ES: Standard effect size
SRM: Standardized response mean

## References

[1] Huang J, Koulaouzidis A, Marlicz W, Lok V, Chu C, Ngai CH, Zhang L, Chen P, Wang S, Yuan J, Lao X-Q, Tse SLA, Xu W, Zheng Z-J, Xie S-H, Wong MCS (2021). Global burden, risk factors, and trends of esophageal cancer: an analysis of cancer registries from 48 countries. Cancers.

[2] Arnold M, Soerjomataram I, Ferlay J, Forman D (2015). Global incidence of oesophageal cancer by histological subtype in 2012. Gut.

[3] Zhang Y (2013). Epidemiology of esophageal cancer. World J Gastroenterol.

[4] Kim TJ, Lee KH, Kim YH, Sung SW, Jheon S, Cho SK, Lee KW (2007). Postoperative imaging of esophageal cancer: what chest radiologists need to know. Radiographics.

[5] Short MN, Aloia TA, Ho V (2014). The influence of complications on the costs of complex cancer surgery. Cancer.

[6] Khullar OV, Jiang R, Force SD, Pickens A, Sancheti MS, Ward K, Gillespie T, Fernandez FG (2015). Transthoracic versus transhiatal resection for esophageal adenocarcinoma of the lower esophagus: a value-based comparison. J Surg Oncol.

[7] Harris A, Bulfone L. Getting value for money: The Australian experience. In: Jost TS (ed) Health care coverage determinations: an international comparative study. Maidenhead: Open University Press, McGraw-Hill International. 2004

[8] National Institute for Health and Care Excellence. Guide to the methods of technology appraisal 2013. In NICE (Ed) 2013. 27905712

[9] Lorgelly PK, Doble B, Rowen D, Brazier J (2017). Condition-specific or generic preference-based measures in oncology? A comparison of the EORTC-8D and the EQ-5D-3L. Qual Life Res.

[10] Teckle P, Peacock S, McTaggart-Cowan H, van der Hoek K, Chia S, Melosky B, Gelmon K (2011). The ability of cancer-specific and generic preference-based instruments to discriminate across clinical and self-reported measures of cancer severities. Health Qual Life Outcomes.

[11] King MT, Costa DS, Aaronson NK, Brazier JE, Cella DF, Fayers PM, Grimison P, Janda M, Kemmler G, Norman R, Pickard AS, Rowen D, Velikova G, Young TA, Viney R (2016). QLU-C10D: a health state classification system for a multi-attribute utility measure based on the EORTC QLQ-C30. Qual Life Res.

[12] Rowen D, Brazier J, Young T, Gaugris S, Craig BM, King MT, Velikova G (2011). Deriving a preference-based measure for cancer using the EORTC QLQ-C30. Value Health J Int Soc Pharmacoecon Outcomes Res.

[13] Brazier J, Ratcliffe J, Salomon AJ, Tsuchiya A. Methods for obtaining health state values: generic preference based measures of health and the alternatives. In Measuring and valuing health benefits for economic evaluation (pp 175–256). New York: Oxford University Press. 2007

[14] Norman R, Viney R, Aaronson NK, Brazier JE, Cella D, Costa DS, Fayers PM, Kemmler G, Peacock S, Pickard AS, Rowen D, Street DJ, Velikova G, Young TA, King MT (2016). Using a discrete choice experiment to value the QLU-C10D: feasibility and sensitivity to presentation format. Qual Life Res.

[15] Rowen D, Young T, Brazier J, Gaugris S (2012). Comparison of generic, condition-specific, and mapped health state utility values for multiple myeloma cancer. Value Health.

[16] Mudge LA, Watson DI, Smithers BM, Isenring EA, Smith L, Jamieson GG (2018). Multicentre factorial randomized clinical trial of perioperative immunonutrition versus standard nutrition for patients undergoing surgical resection of oesophageal cancer. Br J Surg.

[17] EuroQol Group. (2014). EQ-5D.

[18] Aaronson NK, Ahmedzai S, Bergman B, Bullinger M, Cull A, Duez NJ, Filiberti A, Flechtner H, Fleishman SB, de Haes JC (1993). The European Organization for Research and Treatment of Cancer QLQ-C30: a quality-of-life instrument for use in international clinical trials in oncology. J Natl Cancer Inst.

[19] Viney R, Norman R, King MT, Cronin P, Street DJ, Knox S, Ratcliffe J (2011). Time trade-off derived EQ-5D weights for Australia. Value Health.

[20] Gerlinger C, Bamber L, Leverkus F, Schwenke C, Haberland C, Schmidt G, Endrikat J (2019). Comparing the EQ-5D-5L utility index based on value sets of different countries: impact on the interpretation of clinical study results. BMC Res Notes.

[21] Roudijk B, Donders ART, Stalmeier PFM (2019). Cultural Values: Can They Explain Differences in Health Utilities between Countries?. Med Decis Making.

[22] StataCorp. (2017). Stata Statistical Software: Release 15.

[23] Pickard AS, Neary MP, Cella D (2007). Estimation of minimally important differences in EQ-5D utility and VAS scores in cancer. Health Qual Life Outcomes.

[24] Kvam AK, Fayers PM, Wisloff F (2011). Responsiveness and minimal important score differences in quality-of-life questionnaires: a comparison of the EORTC QLQ-C30 cancer-specific questionnaire to the generic utility questionnaires EQ-5D and 15D in patients with multiple myeloma. Eur J Haematol.

[25] Beaton DE, Bombardier C, Katz JN, Wright JG (2001). A taxonomy for responsiveness. J Clin Epidemiol.

[26] Ferguson CJ. An effect size primer: a guide for clinicians and researchers. 2016.

[27] Cohen J (2013). Statistical power analysis for the behavioral sciences.

[28] Taylor TH, Salkind NJ (2010). Ceiling effect. Encyclopedia of research design.

[29] McHorney CA, Tarlov AR (1995). Individual-patient monitoring in clinical practice: are available health status surveys adequate?. Qual Life Res.

[30] Garin O, Michalos AC (2014). Ceiling effect. Encyclopedia of quality of life and well-being research.

[31] Obradovic M, Lal A, Liedgens H (2013). Validity and responsiveness of EuroQol-5 dimension (EQ-5D) versus Short Form-6 dimension (SF-6D) questionnaire in chronic pain. Health Qual Life Outcomes.

[32] Ravens-Sieberer U, Wille N, Badia X, Bonsel G, Burström K, Cavrini G, Devlin N, Egmar AC, Gusi N, Herdman M, Jelsma J, Kind P, Olivares PR, Scalone L, Greiner W (2010). Feasibility, reliability, and validity of the EQ-5D-Y: results from a multinational study. Qual Life Res.

[33] Bland JM, Altman D (1986). Statistical methods for assessing agreement between two methods of clinical measurement. The lancet.

[34] Bland JM, Altman DG (2010). Statistical methods for assessing agreement between two methods of clinical measurement. Int J Nurs Stud.

[35] Tordrup D, Mossman J, Kanavos P (2014). Responsiveness of the EQ-5D to clinical change: is the patient experience adequately represented?. Int J Technol Assess Health Care.

[36] Brazier JE, Harper R, Munro J, Walters SJ, Snaith ML (1999). Generic and condition-specific outcome measures for people with osteoarthritis of the knee. Rheumatology (Oxford).

[37] Krabbe PFM, Peerenboom L, Langenhoff BS, Ruers TJM (2004). Responsiveness of the generic EQ-5D summary measure compared to the disease-specific EORTC QLQ C-30. Qual Life Res.

[38] Solberg TK, Olsen J-A, Ingebrigtsen T, Hofoss D, Nygaard ØP (2005). Health-related quality of life assessment by the EuroQol-5D can provide cost-utility data in the field of low-back surgery. Eur Spine J.

[39] Krahn M, Bremner KE, Tomlinson G, Ritvo P, Irvine J, Naglie G (2006). Responsiveness of disease-specific and generic utility instruments in prostate cancer patients. Qual Life Res.

[40] Kaarlola A, Pettila V, Kekki P (2004). Performance of two measures of general health-related quality of life, the EQ-5D and the RAND-36 among critically ill patients. Intensive Care Med.

[41] Kim SH, Kim HJ, Lee SI, Jo MW (2012). Comparing the psychometric properties of the EQ-5D-3L and EQ-5D-5L in cancer patients in Korea. Qual Life Res.

[42] Chen G, Olsen JA (2020). Filling the psycho-social gap in the EQ-5D: the empirical support for four bolt-on dimensions. Qual Life Res.

[43] Herdman M, Gudex C, Lloyd A, Janssen M, Kind P, Parkin D, Bonsel G, Badia X (2011). Development and preliminary testing of the new five-level version of EQ-5D (EQ-5D-5L). Qual Life Res Int J Qual Life Aspects Treat Care Rehabil.

[44] Gamst-Klaussen T, Chen G, Lamu AN, Olsen JA (2016). Health state utility instruments compared: inquiring into nonlinearity across EQ-5D-5L, SF-6D, HUI-3 and 15D. Qual Life Res.

[45] Dakin H (2013). Review of studies mapping from quality of life or clinical measures to EQ-5D: an online database. Health Qual Life Outcomes.

[46] McTaggart-Cowan H, Teckle P, Peacock S (2013). Mapping utilities from cancer-specific health-related quality of life instruments: a review of the literature. Expert Rev Pharmacoecon Outcomes Res.

[47] Brazier J, Ratcliffe J, Saloman J, Tsuchiya A. Alternatives to generic preference-based measures: mapping, condition-specific measures, bolt-ons, vignettes, direct utility assessment, and well-being. In: Measuring and valuing health benefits for economic evaluation (2nd ed) Oxford: Oxford University Press. 2016.

