# Supplementary material for: Responsiveness and convergent validity of QLU-C10D and EQ-5D-3L in assessing short-term quality of life following esophagectomy
Source: Health Qual Life Outcomes. 2021 Oct 2;19:233. doi: 10.1186/s12955-021-01867-w (PMC8487554; doi:10.1186/s12955-021-01867-w)

**Supplementary material**

# Title: Responsiveness and convergent validity of QLU-C10D and EQ-5D-3L in assessing short-term quality of life following esophagectomy

## Comparing demographics and quality of life outcomes for excluded respondents

The analysis in this paper only included patients with both baseline (pre-operative) and follow-up (post-operative) quality of life scores on all instruments. Tables A1 and A2 show differences in demographic characteristics and quality of life outcomes for patients included and those excluded from the analysis.

**Table A1 Comparing Demographic characteristics for excluded respondents**

| **Characteristic** | **Total sample** | **Complete QoL data (n=164)** | **Incomplete QoL data** | **p-value** |
| --- | --- | --- | --- | --- |
| **Continuous variables [Mean (sd)]** | | | | |
| Age | 63.5 (8.7) | 62.9 (7.9) | 64.4 (9.6) | 0.06 |
| Hospital length of stay (days) | 18.7 (17.1) | 17 (15) | 21.8 (20.1) | 0.08 |
| ICU length of stay (days) | 4.7 (8) | 4 (4) | 5.8 (11.7) | 0.66 |
| Blood loss (ml) | 263.4 (244.9) | 268.1 (256.1) | 255.5 (225.9) | 0.89 |
| Blood transfusion (units) | 0.28 (1.61) | 0.31 (1.98) | 0.22 (0.81) | 0.89 |
| Tumour length (cm) | 3.62 (2.62) | 3.86 (2.66) | 3.24 (2.52) | 0.14 |
| Total pack years | 17.9 (20.2) | 16 (18) | 20.1 (23.4) | 0.50 |
| **Categorical variables** | **N (%)** | | |  |
| Male Gender | 223 (81) | 133 (81) | 90 (80) | 0.88 |
| Hospital length of stay > 10 days | 213 (81) | 133 (81) | 80 (80) | 0.83 |
| Tumour length >3cm | 87 (47) | 58 (51) | 29 (40) | 0.16 |
| **Co-morbidities** |  |  |  |  |
| Hypertension | 105 (39) | 62 (38) | 43 (41) | 0.61 |
| Diabetes | 35 (13) | 21 (13) | 14 (13) | 0.92 |
| Respiratory | 61 (22.6) | 33 (20) | 28 (26) | 0.23 |
| Cardiac | 52 (19) | 27 (17) | 25 (24) | 0.15 |
| Alcohol | 164 (62) | 106 (66) | 58 (56) | 0.1 |
| Smoking | 36 (13) | 22 (13) | 14 (13) | 0.96 |
| Preoperative Radiotherapy | 112 (41) | 67 (41) | 45 (42) | 0.8 |
| Preoperative Chemotherapy | 211 (78) | 129 (79) | 82 (77) | 0.80 |
| **ASA score** |  |  |  |  |
| 1 or 2 | 178 (67) | 111 (68) | 67 (66) | 0.77 |
| 3 | 86 (33) | 52 (32) | 34 (34) |  |
| **Pathological T staging** |  |  |  |  |
| 0/1/1a/1b/Tis | 112 (43) | 72 (44) | 40 (41) | 0.67 |
| 2/3 | 149 (57) | 92 (56) | 57 (59) |  |
| **TNM staging** |  |  |  |  |
| 0/IA/IB | 112 (43) | 75 (46) | 37 (37) | **0.01** |
| II/IIB/IIIA/IIIB | 130 (49) | 82 (50) | 48 (48) |  |
| IIIC/4 | 21 (8) | 7 (4) | 14 (14) |  |
| **Procedure type** |  |  |  |  |
| Open chest and abdominal approach | 108 (42) | 76 (46) | 32 33.68 | **0.05** |
| Hybrid (thoracoscopic) | 151 58.30 | 88 (54) | 63 66.32 |  |

**Table A2 Comparing Quality of life outcomes for excluded respondents^1^**

| **Quality of life measure** | **Total sample** | **Complete QoL data** | **Incomplete QoL data** | **p-value** |
| --- | --- | --- | --- | --- |
| **Baseline (7 days before esophagectomy)** | | | | |
| EQ-5D-3L | 0.85 (0.15) | 0.85 (0.15) | 0.84 (0.15) | 0.71 |
| QLU-C10D | 0.80 (0.17) | 0.81 (0.16) | 0.80 (0.18) | 0.90 |
| EORTC summary score | 84.3 (12.7) | 84.3 (13.1) | 84.6 (11.9) | 0.90 |
| **Follow – up (42 days after esophagectomy)** | | | | |
| EQ-5D-3L | 0.69 (0.18) | 0.69 (0.16) | 0.69 (0.24) | 0.15 |
| QLU-C10D | 0.52 (0.22) | 0.52 (0.22) | 0.53 (0.24) | 0.50 |
| EORTC summary score | 62.4 (18.5) | 62.5 (17.9) | 61.9 (21.0) | 0.83 |

^1^The analysis in this paper only included patients with both baseline (pre-operative) and follow-up (post-operative) quality of life scores on all instruments. This table shows differences in quality of life for patients included and those excluded from the analysis. There were no differences in quality of life between the two groups

## Additional files on Complete case analysis

### Distribution of QoL scores for each instrument (complete cases)

**Fig A1 Baseline QoL (7 days before esophagectomy)**


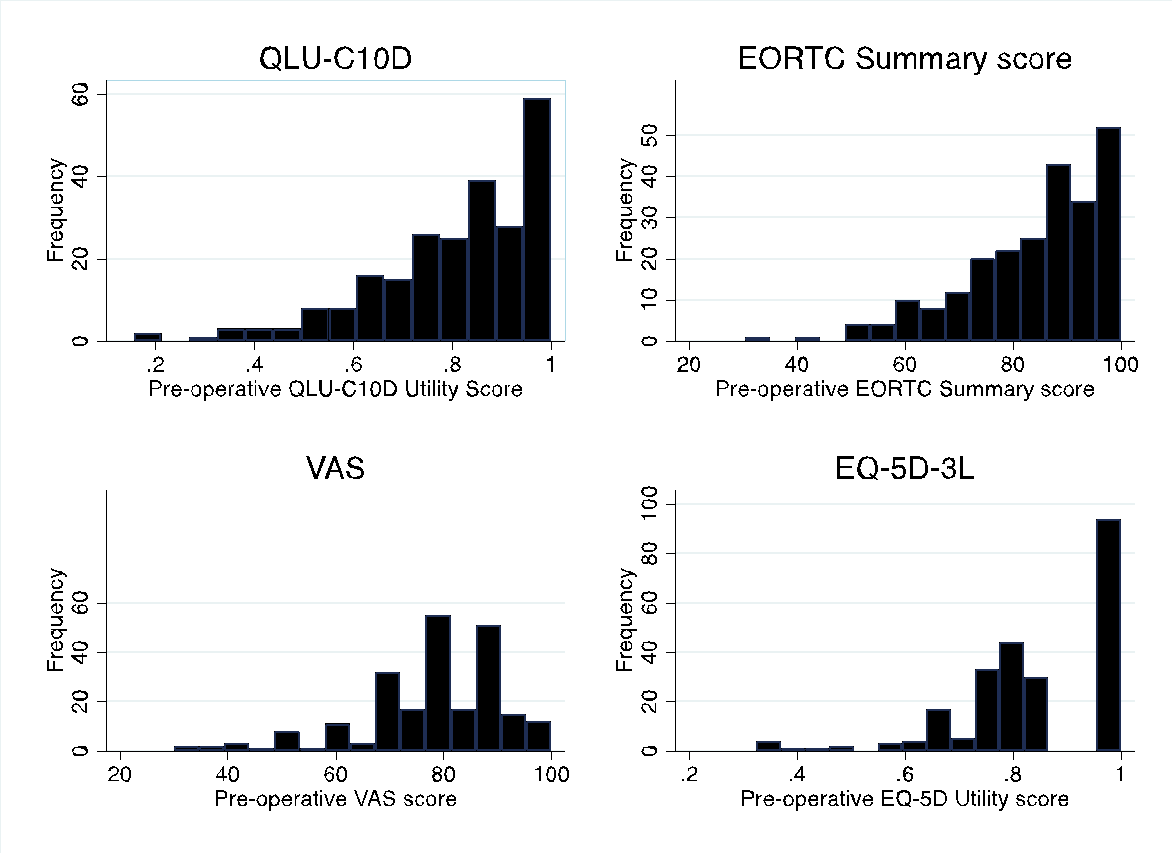


**Fig A2 Follow-up QoL (42 days after esophagectomy)**


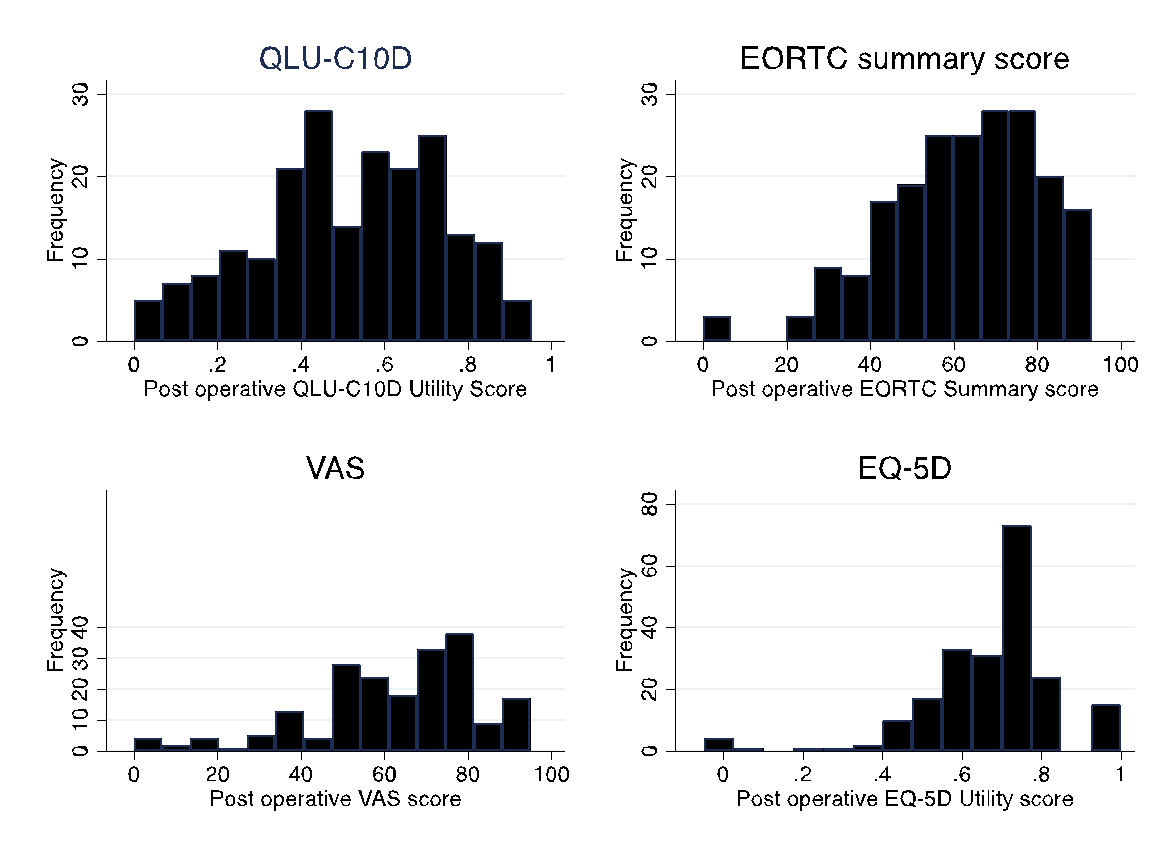


**Fig A3 Distribution of the mean difference in QoL scores for each instrument**


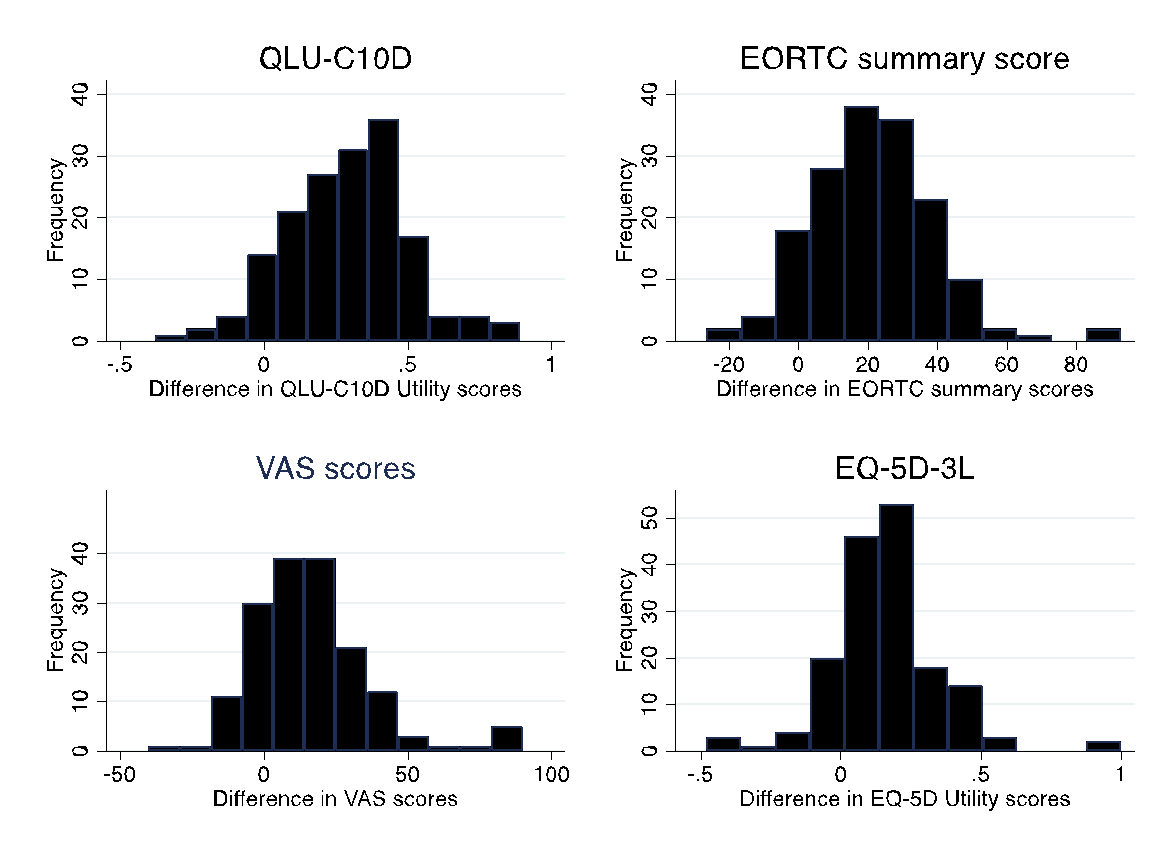

Supplement: Supplementary file 1 — Additional file 1. Supplementary material [file 12955_2021_1867_MOESM1_ESM.docx]
